# KU60019 inhibits ovarian cancer progression by targeting DGAT1/has-miR-1273g-3p axis

**DOI:** 10.1371/journal.pone.0325213

**Published:** 2025-06-24

**Authors:** Zhanchuan Ma, Rongjing Dang, Guodong Wu

**Affiliations:** 1 Central Laboratory, Lequn Branch, The First Hospital of Jilin University, Changchun, Jilin, China; 2 Department of Cardiology, Lequn Branch, The First Hospital of Jilin University, Changchun, Jilin, China; McGill University, CANADA

## Abstract

Ataxia telangiectasia mutated (ATM) blockage can induce apoptosis in ovarian cancer. However, the molecular mechanisms underlying this process remain poorly understood. In this study, ovarian cancer cells (SKOV3) were treated with an ATM inhibitor (KU60019) for 24 hours, and the fold changes of DGAT1 and hsa-miR-1273g-3p were quantified by real-time quantitative polymerase chain reaction (RT-qPCR). Gene Ontology (GO) and pathway enrichment analyses of DGAT1-associated functions were performed. Hsa-miR-1273g-3p mimics were used to investigate the relationship between DGAT1 and hsa-miR-1273g-3p in ovarian cancer cells under ATM inhibitor treatment, and cell apoptosis rate, viability, and migration were detected. The DGAT1 inhibitor reversed KU60019-induced migration impairment in SKOV3 cells. Finally, Kaplan-Meier analysis showed the correlation between DGAT1 level and survival in ovarian cancer patients. We found that ATM blockage significantly suppressed hsa-miR-1273g-3p level and elevated DGAT1 level in SKOV3 cells. DGAT1 was enriched in cytokine receptor interaction, T cell receptor signaling pathway, and cell apoptosis. Hsa-miR-1273g-3p mimics reversed suppression of DGAT1 and impaired cell viability induced by KU60019. Higher levels of DGAT1 associated with worse survival in ovarian cancer patients. KU60019 induced ovarian cancer cell impairment by enhancing DGAT1 level and suppressing hsa-miR-1273g-3p level. Our results demonstrate the antitumor effect of KU60019 in ovarian cancer depended on miR1273g-3p/DGAT1 axis.

## Introduction

Rapidly increasing incidence of ovarian cancer and high mortality rate make it the leading cause of death from gynecologic cancer [1]. Although chemotherapy, surgical excision, and biotherapy are used to maximally reduce the physical burden of cancer patients, the five-year survival rate remains poor, primarily because most ovarian cancer patients are diagnosed at an advanced stage [1]. Ovarian cancer could occur at all ages, and current therapeutic strategies typically involve a combination of chemotherapy, radiotherapy, and biotherapy, which are closely linked to advances in understanding the molecular mechanisms underlying antitumor therapies [2,3]. Targeting key genes to improve anti-tumor therapy has been recognized as a promising approach for eliminating cancer cells.

Ataxia telangiectasia mutated (ATM) is a key protein belonging to the phosphatidylinositol 3-kinase-related kinase family, involved in the DNA repair system [4]. ATM gene mutations or low ATM expression in cancer cells lead to reduced cell migration, **upregulated autophagy**, and enhanced sensitivity to chemotherapy and radiotherapy [5,6]. Phosphorylation of ATM autophosphorylation sites (Ser367, Ser1893, Ser1981, Ser2996, and Thr1885) triggers the initiation of DNA damage repair [6]. Selective ATM kinase inhibitor like KU60019 could effectively reduce cancer cell survival by disturbing DNA repair system. Administration of KU60019 enhanced sensitivityto KMT2A-rearranged acute leukemias [7]. Combining KU60019 with Type II topoisomerase (TOP2) poisons accelerates cell death via suppression of DNA damage response in lung cancer cell lines [8]. ATM inhibition is also effective in the treatment of ovarian cancer [9], targeting ATM might be a potential strategy for anti-cancer therapy.

Diacylglycerol acyltransferase 1 (DGAT1), a key enzyme involved in regulating triacylglycerol accumulation, which has recently been recognized as a key enzyme in triacylglycerols biosynthesis and lipid droplet formation [10]. Accumulation of triglyceride is involved in DNA damage and ATM pathway in monocytes [11]. Mice deficient in DGAT1 exhibit resistance to diet-induced obesity [12]. Elevated DGAT1 expression was reported to be correlated with worse survival in gastric cancer patients [13,14], and is significantly associated with advanced cancer stage and survival in ovarian cancer patients [15]. The influence of DGAT1 on anti-tumor therapy is currently under investigation. DGAT1 inhibition restricts lipid metabolism, leading to increased ROS release in glioblastoma, and subsequent suppression of tumor growth [10,16]. DGAT1 inhibitors significantly suppressed cleaved caspase-3 (the key executioner of apoptosis) in endometrial cancer [17], indicating an important role for DGAT1 in regulating cell apoptosis. Targeting DGAT1 is an effective strategy for tumor treatment [10]. However, the role of DGAT1 in ovarian cancer during ATM inhibition remains poorly understood.

In the current study, we hypothesized that blocking DGAT1 might inhibit tumor growth, and investigated the role of DGAT1 in ovarian cancer cells in the context of ATM blockage *in vitro*. We identified DGAT1 as a potential target of miR-1273g-3p in ovarian cancer cell under KU60019 treatment. Our results revealed cellular and molecular pathways in KU60019-induced cell death as well as revealed novel therapeutic targets for anti-tumor research.

## Materials and methods

### Bioinformatics data mining

Original expression data of the target gene in ovarian cancer tissues were obtained from The Cancer Genome Atlas (TCGA) database, and analyzed by the online database GEPIA [18] (http://gepia.cancer-pku.cn/about.html). We used the online tool Kaplan-Meier Plotter [19] (http://kmplot.com/analysis/index.php?p=background) to investigate the correlation between the target gene and overall survival (OS), progression-free survival (PFS), and postprogression survival (PPS) in patients with ovarian cancer. Tumor IMmune Estimation Resource [20] (https://cistrome.shinyapps.io/timer/) was used to investigate the role of target gene in tumor infiltrates immune cells. TargetScan Release 8.0 [21] (https://www.targetscan.org/vert_80/) was used to predict the binding site between the target gene and microRNA. KOBAS (http://kobas.cbi.pku.edu.cn/kobas3/genelist/) was used to investigate the enrichment terms of the target gene. No human participants or tissues were involved in current study.

### Cell culture and treatment

Ovarian carcinoma cell line (SKOV3) and human renal epithelial cell line (293FT) were purchased from the Shanghai Zhong Qiao Xin Zhou Biotechnology Co., Ltd, and cultured in Dulbecco’s Modified Eagle Medium supplemented with 10% fetal calf serum, 100 U/ml penicillin, and 100 ug/ml streptomycin. ATM inhibitor KU60019 and DGAT1 inhibitor (DGAT1i) A922500 were purchased from the Topscience Co. Ltd. miRNA (miRNA-negative control and miR-1273g-3p mimics) was purchased from Shanghai GenePharma Co., Ltd. KU60019 (10 mM) and DGAT1i (100 mM) was dissolved in dimethylsulfoxide and stored at −80°C under sterile conditions. Cells were treated with 10nM KU60019, or 200uM DGATi, or 20 nM miR-1273g-3p mimics for 24 hours as previously described [9,22]. For cell transfection, ovarian cancer cells in a 6-well plate with 70% confluence were transfected with a negative control or miR-1273g-3p mimics. All cells were maintained in a humidified CO^2^ (5%) incubator at 37°C.

### Cell apoptosis analysis

The cells were digested with 0.25% trypsin for 1 minute, then the cells were washed twice with phosphate-buffer saline, collected and stained using the Annexin V-FITC/PI Apoptosis Detection Kit (Yeasen Biotechnology (Shanghai) Co., Ltd) according to the manufacturer’s protocols. Apoptotic ratio was determined using a Ariall flow cytometer (BD Biosciences).

### RNA extraction and quantitative real-time PCR

Total RNA was extracted using the MolPure® Cell/Tissue Total RNA Kit (Yeasen Biotechnology (Shanghai) Co., LTD). DGAT1 cDNA was synthesized using Hifair® Ⅱ 1st Strand cDNA Synthesis Kit (Yeasen Biotechnology (Shanghai) Co., LTD), TransScript® miRNA RT Enzyme Mix (TransGen Biotech Co., Ltd.) was used for synthesis of first-strand cDNA of miR-1273g-3p according to the manufacturer’s protocols. qRT-PCR was performed on ABI StepOnePlus system (Applied Biosystems) with TB Green Fast qPCR Mix (Takara Biomedical Technology (Beijing) Co., Ltd.). Expression of miR-1273g-3p was normalized against U6, and DGAT1 expression was normalized to GAPDH expression. The primer sequence sets were listed as follows:

Primers for target genes

**Table:** 

Primer name	Primer sequence
*GAPDH* [23]	F: 5’ *CTGGGCTACACTGAGCACC* 3’ R: 5’ *AAGTGGTCGTTGAGGGCAATG* 3’
*DGAT1* [24]	F: 5’ TATTGCGGCCAATGTCTTTGC 3 R: 5’CACTGGAGTGATAGACTCAACCA 3’
*U6* [25]	F: 5’ CGCTTCGGCAGCACATATAC 3 R: 5’ TTCACGAATTTGCGTGTCATC 3’

### Cell transfection

Before DGAT1i or miRNA-1273g-3p mimics treatment, cells were treated with KU60019 for 24 hours. Then, miRNA-1273g-3p mimics and **negative control (NC)** were transfected into SKOV3 cells with the Lipofectamine RNAiMAX Transfection Reagent (Thermo Fisher Scientific Inc.). Transfected cells were cultured in RPMI-1640 medium for further studies.

### CCK-8 cell viability assay

SKOV3 cells or 293FT cells transfected with sh-Ctrl and miR-1273g mimics, or treated with KU60019 were seeded in 96-well plates at a density of 2000 cells/well. Then, Cell Counting Kit (Yeasen Biotechnology (Shanghai) Co., Ltd.) was added to indicated wells for 30 mins according to the manufacturer’s protocol. Cell viability was determined by recording absorbance at 450 nm [26] using Synergy HT microplate reader (BioTek, USA).

### Wound healing assay

Cells treated with DGAT1i or KU60019 in 6-well plates were scraped straightly using a 200 ul pipette tip upon reaching approximately 95% confluence, then unadherent cells were removed by washing with phosphate buffer saline. Random images were taken immediately at 0 and 24 hours after scraping. The relative wound area (%) was quantified by Image J software.

### Dual-luciferase reporter assay

The luciferase assay was performed as previously described to verify the relationship between hsa-miRNA-1273g-3p and the 3'-UTR of DGAT. HEK-293T cells were transfected with the pGL3 luciferase vector with the wild‐type or mutated DGAT1‐untranslated region, with or without the miR-1273g-3p negative control (NC) or miR-1273g-3p mimics (Genepharma, China) for 48 hours. Cell lysates were collected and firefly and Renilla luciferase activities were detected using a standard multimode plate reader (Biotek Epoch, USA).

### Statistical analysis

Statistical analyses were performed using GraphPad Prism software version 7. Significant differences between two groups were analyzed by two-tailed Student’s t-tests. *p* value of less than 0.05 was considered statistically significant. Quantification of signal was shown in bar graphs and error bars represent mean ± SD.

## Results

### ATM inhibition triggered apoptosis in ovarian cancer cells

**Fig 1:**
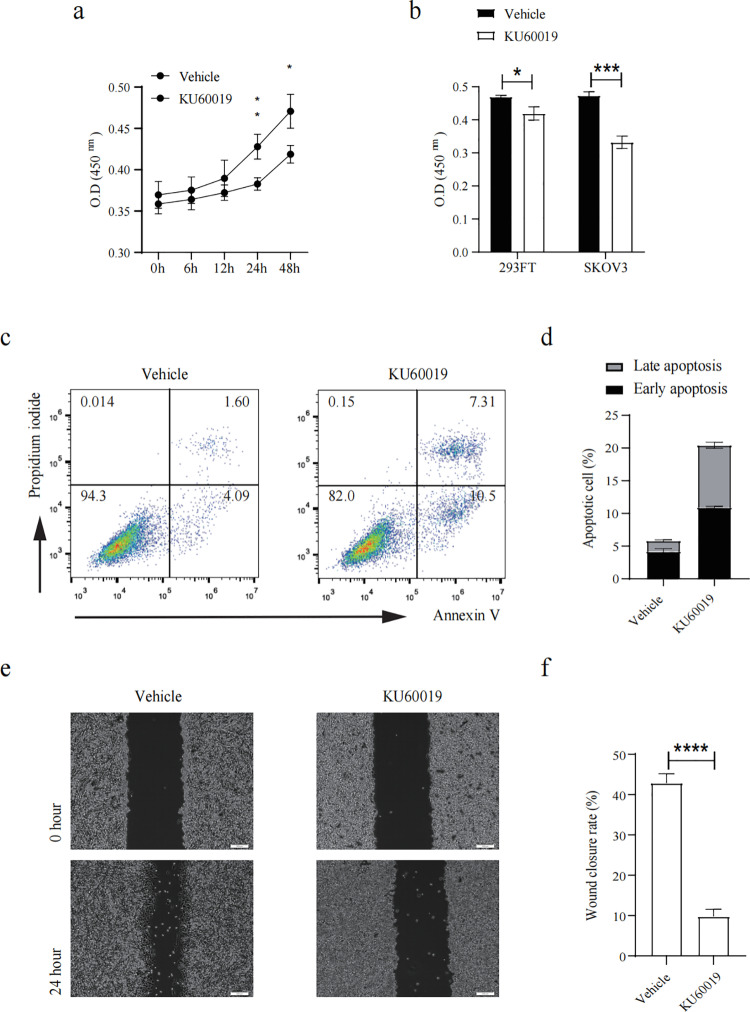
ATM blockage induced apoptosis in ovarian cancer cell. SKOV3 cells or 293FT cells were treated with KU60019 for up to 48 hours, CCK-8 assay was performed to investigate the cell viability (a-b), cells were imaged at 0 hour and 24 hours. Cell apoptotic rate was detected by flow cytometry(c-d). Cell migration area was assessed by the Image J software (e-f). (****p < 0.0001).

Cell viability was measured at different time points after further stimulation with 20 nM ATM inhibitor KU60019. Results showed that the drug treatment for 24 hours significantly reduced cell viability (Fig 1a), and the effect on tumor cells was greater than that on non-tumor cells (Fig 1b). In addition, a significantly increased early apoptosis ratio (Annexin V+ propidium iodide- cells) in ovarian cancer cells was observed (Fig 1c, d), and cell ratio in late apoptosis also had a remarkable accumulation (Fig 1c, d). Cell migration was significantly restrained after KU60019 treatment (Fig 1e, f). These results indicated a therapeutic potential of KU60019 in ovarian cancer cell treatment.

### KU60019 treatment upregulated DGAT1 expression in ovarian cancer cells

**Fig 2:**
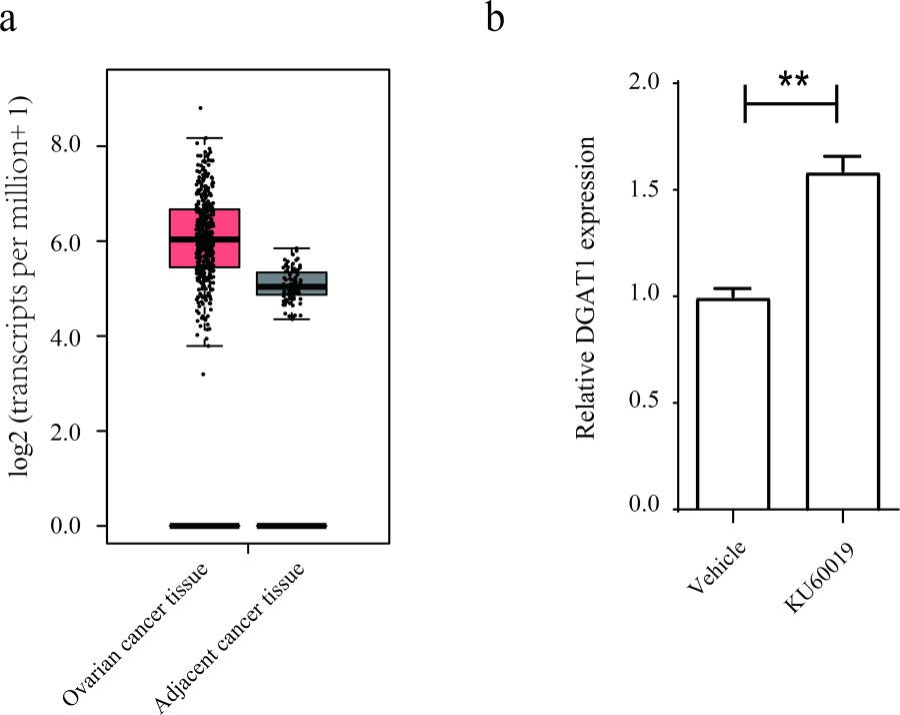
KU60019 enhanced DGAT1 expression in ovarian cancer cell. (a) Expression of DGAT1 in ovarian cancer tissues, data was collected from the TCGA database (tumor tissues, n = 426; adjacent tissues, n = 88). (b) The level of DGAT1 in SKOV3 cells were determined using qPCR. (**p < 0.01).

Elevated DGAT1 levels in ovarian cancer tissues show positive correlation with advanced cancer stages [15]. However, the regulatory effect of KU60019 on DGAT1 expression in ovarian cancer cells remains poorly characterized. Analysis of TCGA ovarian cancer datasets confirmed DGAT1 overexpression in tumor tissues compared to normal controls (Fig 2a, S1 Fig). Unexpectedly, we found that KU60019 treatment leaded to overexpression of DGAT1 in ovarian cancer tissues, but not in non-tumor cells (Fig 2b, S1 Fig). Gene ontology analysis revealed significant enrichment of DGAT1 in apoptosis-related pathways (S1 Table). These findings suggest that ATM inhibition-induced apoptosis in ovarian cancer cells may be mechanistically linked to DGAT1 dysregulation.

### KU60019 treatment triggered concurrent downregulation of hsa-miR-1273g-3p and upregulation of DGAT1 expression in ovarian cancer cells

**Fig 3:**
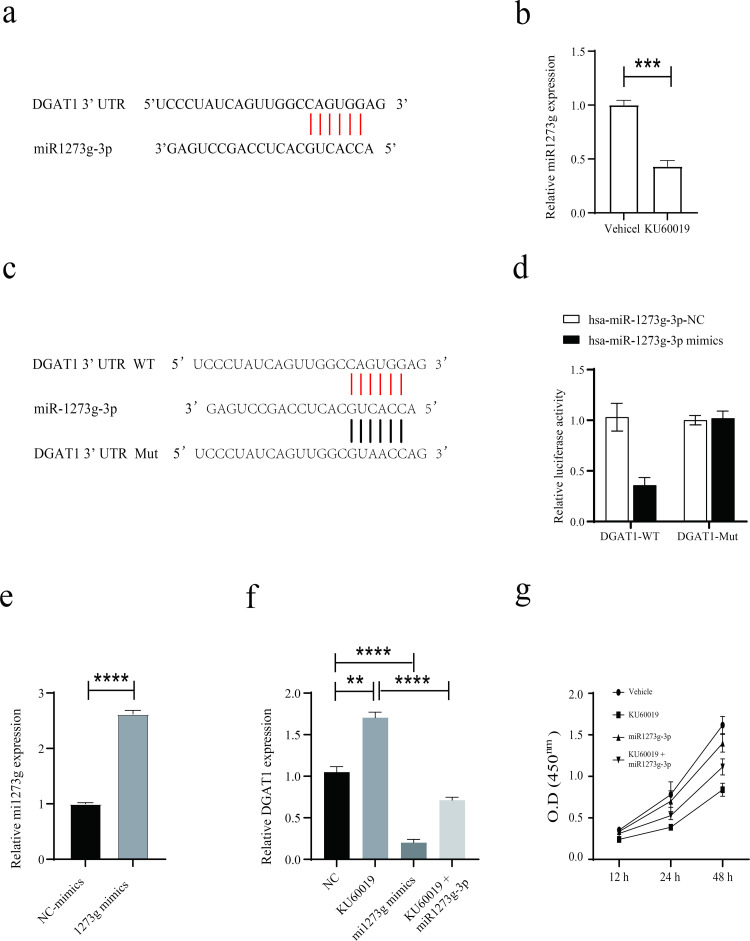
DGAT1 was a potential target of miR-1273g-3p. (a) Sequences of the predicted binding sites between miR-1273g-3p and the 3′-UTR of the DGAT1. KU60019-treated SKOV3 cells were transfected with miR-1273g-3p mimics or negative control for 24 hours. For luciferase reporter assay, the luciferase activity of pGL3-DGAT1-UTR-WT or pGL3-DGAT1-UTR-Mut in 293FT cells after co-transfection with negative control for miR-1273g-3p or miR-1273g-3p mimics, absorbance at 450 nm was recorded (c-d). The expression of miR-1273g-3p (b, e), and DGAT1 (f) were determined using qPCR and CCK8 assay (e). (*p < 0.05; **p < 0.01; ***p < 0.001; ****p < 0.0001).

Next, we sought to investigate the mechanism underlying KU60019-mediated suppression of DGAT1 expression. As results from the TargetScan 8.0 showed that hsa-miR-1273g-3p (miR-1273g-3p) could potentially interact with the 3′ untranslated regions (UTR) of DGAT1 mRNAs, (Fig 3a), and targets of miR-1273g-3p were enriched in disease-related pathways and transcription factors (S2 Fig), based on these findings, we quantified the level of miR-1273g-3p in ovarian cancer cells. KU60019 treatment significantly reduced miR-1273g-3p expression, with stronger suppression observed in tumor cells compared to non-tumor counterparts (Fig 3b, S3 Fig). The luciferase activity of pGL3 luciferase reporter gene was reduced after co-transfection with pGL3-DGAT1-UTR-WT and miR-1273g-3p mimics, but it showed no statistically significant difference after co-transfection with pGL3-DGAT1-UTR-Mut and miR-1273g-3p (Fig 3c, d), confirmed a direct interaction between DGAT1 and miR-1273g-3p. Meanwhile miR-1273g-3p mimics enhanced miR-1273g-3p level in ovarian cancer cells (Fig 3e). Notably, the KU60019-induced suppression of DGAT1 was partially rescued upon miR-1273g-3p mimic transfection (Fig 3f). Furthermore, miR-1273g-3p mimics attenuated the KU60019-induced reduction in cell viability (Fig 3g). Collectively, these findings indicate that miR-1273g-3p mediates DGAT1-regulated apoptotic processes in ovarian cancer cells upon KU60019 treatment.

### Pharmacological inhibition of DGAT1 significantly rescued the KU60019-induced suppression of migratory capacity in SKOV3 ovarian cancer cells

**Fig 4:**
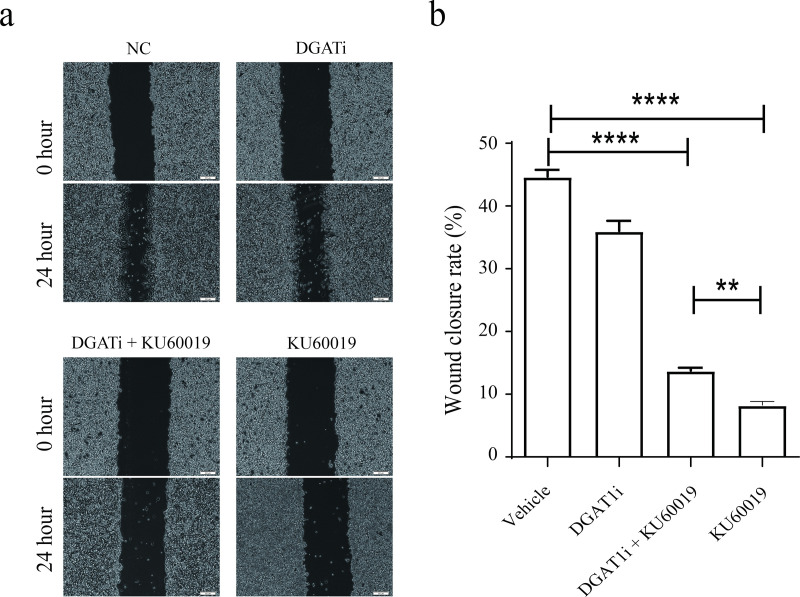
DGAT1 inhibitor reversed KU60019 induced migration impairment in SKOV3 cells.

**Fig 5:**
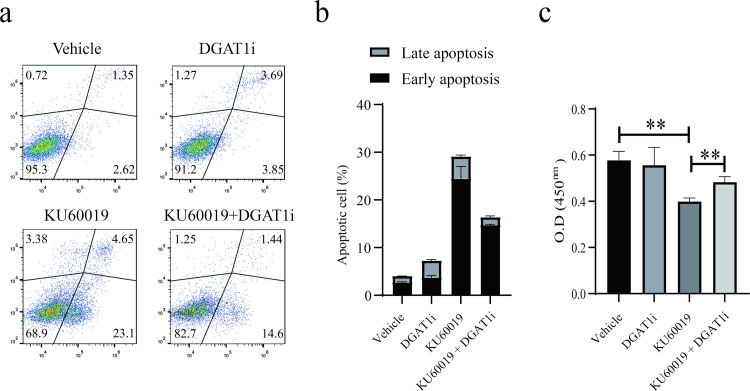
Inhibition of DGAT1 significantly alleviated KU60019-induced cell apoptosis. KU60019-treated SKOV3 cells were treated with DGAT1 inhibitor or negative control for 24 hours. Cells were collected for apoptotic rate detection (a-b) and viability detection (c). (**p < 0.01).

To confirm the role of DGAT1 in ovarian cancer cells, cell migration ratio was accessed after administration of DGAT1 inhibitor for 24 hours. KU60019 treatment markedly impaired SKOV3 cell migration, an effect substantially reversed through DGAT1 inhibition, and the effect on tumor cells was greater than that on non-tumor cells (Fig 4a,b). However, the same treatment had little effect on non-tumor cells (S4 Fig). Mechanistically, DGAT1 inhibition attenuated KU60019-induced apoptosis and partially restored cellular viability (Fig 5a–c). These findings establish DGAT1 as a critical mediator of KU60019’s therapeutic effects in ovarian cancer pathogenesis. Unexpectedly, we found that KU60019 treatment led to overexpression of DGAT1 in ovarian cancer tissues, but not in non-tumor cells

Cells with above 95% confluence in the 6-well plate were used in the following experiment. KU60019-treated SKOV3 cells were treated with DGAT1 inhibitor or negative control for 24 hours. Cells were imaged at 0 hour and 24 hours to assess the migration ability (a-b). (**p < 0.01; ****p < 0.0001).

### Elevated DGAT1 expression served as an independent prognostic factor for reduced overall survival in ovarian cancer patients

**Fig 6:**
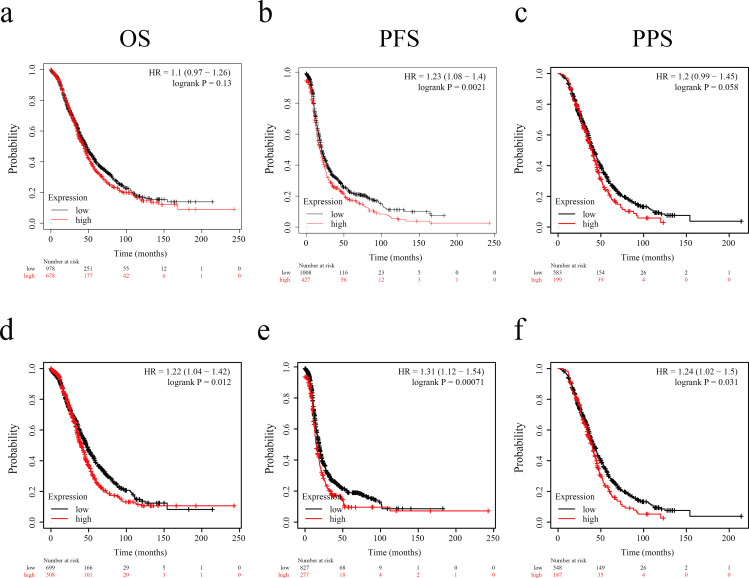
Aberrant expression of DGAT1 significantly correlates with poor prognosis of ovarian cancer patients. Kaplan–Meier survival analysis with the log-rank test was used to determine the association of DGAT1 with OS (a), PFS (b), and PPS (c) in 1656 ovarian cancer patients. Correlation between the level of DGAT1 and OS (d), PFS (e), and PPS (f) in 1207 serous ovarian cancer patients were investigated.

We investigated the relationship between DGAT1 and survival of ovarian cancer patients based on level of DGAT1 in ovarian cancer tissues (Red, high expression; black, low expression). While DGAT1 expression showed no significant association with overall survival (Fig 6a), elevated DGAT1 levels correlated with reduced progression-free survival (PFS; Fig 6b). A non-significant trend was observed between increased DGAT1 expression and poorer post-progression survival (PPS; Fig 6c). However, in the serous histology subgroup, elevated DGAT1 expression demonstrated significant negative correlations with overall survival (OS; Fig 6d), progression-free survival (Fig 6e), and post-progression survival (Fig 6f). Significant correlation was observed between DGAT1 expression levels and tumor-infiltrating CD4^+^ T lymphocyte density (S5 Fig). These findings position DGAT1 as a promising prognostic biomarker in ovarian cancer.

## Discussion

DNA damage response could be initiated by the ataxia telangiectasia-mutated kinase (ATM), phosphorylation of ATM leads to cell cycle arrest: ameliorated DNA damage give rise to DNA repair, otherwise result in apoptosis [27]. In current study, we found that the expression of DGAT1 was significantly suppressed in ovarian cancer cell line SKOV3 cells after ATM inhibitor KU60019 treatment. In the search for mechanism of DGAT1 inhibition, KU60019 treatment upregulated the level of miR-1273g-3p in SKOV3 cells, which could directly bind to DGAT1, leading to impairment of cell viability. In addition, we observed high level of DGAT1 in ovarian cancer tissues predicted a poor outcome in ovarian cancer patients. Our results demonstrated that cellular damage caused by ATM inhibition depends on miR-1273g-3p/DGAT1 pathway.

Recent studies have shown that circulating miR-1273g-3p in pancreatic cancer predicted negative predictive value in patients, and increased miR-1273g-3p level also associated with tumor stage in pancreatic cancer patients [28]. It was seemed that high level of miR-1273g-3p promoted cancer progression. However, our results showed that high level of miR-1273g-3p could directly bind to DGAT1, leading to impaired cell migration ability *in vitro*, which indicated that in different tumor microenvironments, miR-1273g-3p might bind to different targets that expressed at high levels to play a complex role in regulating cancer cell progression. Another group showed that high level of miR-1273g-3p promotes autophagosomes-Lysosomal fusion and reduces the accumulation of SQSTM1/p62 protein, protectively reduces Aβ1–42-induced neuronal apoptosis [29], indicated a crucial role of miR-1273g-3p in disease development.

MicroRNAs (miRNAs) mediate post-transcriptional gene silencing through direct binding to target mRNAs. Here, we predicted that DGAT1 was target gene of miR-1273g-3p. DGAT1 synthesize triglycerides by transferring acyl-CoA to diacylglycerol, which plays a crucial role in lipid droplet biogenesis, thus, promote fatty acids absorption [30]. It was reported that overexpression of DGAT1 in ovarian cancer tissues predicted a worse outcome in ovarian cancer patients, suppression of DGAT1 reduced cell proliferation and migration [15]. Our results showed that the function of KU60019 in regulating of cell apoptosis was through the miR-1273g-3p/DGAT1, in accordance with the observation of alleviated cell injury in miR-1273g-3p suppression group. In addition, we showed that blockage of DGAT1 alleviated cell apoptosis during KU60019 treatment. It seems that the regulatory effect of DGAT1 on tumor progression involves many aspects. For example, ionizing radiation increased DGAT1 expression in glioblastoma, genetic inhibition of DGAT1 suppresses radioresistance and enhanced radiosensitivity of U87MG and U87MG-RR cells [31]. Upregulation of DGAT1 could also be observed in melanoma, which was correlated with reduced patient survival, selective DGAT1 inhibitors suppressed lipid droplets formation and suppressed melanoma cell growth [32]. In this study, we found that higher expression of DGAT1 associated with poor survival in ovarian cancer patients, progression-free survival and postprogression survival could also be impacted by DGAT1 expression in patients. In tumor tissue rich in the DGAT1 gene displayed raised survival was related with CD4 + T cells and dendritic cell. Indeed, GSEA analysis showed that DGAT1 may be involved in the immune process [15].

In conclusion, our study elucidates a novel mechanism underlying KU60019-induced ovarian cancer cell death through the miR-1273g-3p/DGAT1 regulatory axis. We demonstrate that KU60019-mediated remodeling coordinates miR-1273g-3p upregulation and subsequent DGAT1 suppression, ultimately impairing cancer cell migration and inducing apoptosis. These findings collectively establish DGAT1 as a critical mediator of therapeutic response to ATM inhibition and uncover its potential as a druggable target for ovarian cancer treatment.

## Supporting information

S1 Table KEGG analysis of DGAT1. (DOCX)

S1 Fig ATM blockage elevated the DGAT1 expression in SKOV3 cells. (a)The proportion of patients with different cancer stages. (G1, stage 1; G2, stage 2; G3, stage 3; G4, stage 4; GX, undetermined grade. n = 587). (b)The proportion of ovarian cancer patients in different age groups. Data was obtained from the TCGA. (n = 587). (c)SKOV3 cells or 293FT cells were treated with KU60019 for 24 hours, the level of DGAT1 in cells were determined using qPCR. (*p < 0.05; **p < 0.01).
(TIF)

S2 Fig Summary of enrichment analysis of hsa-miR-1273g-3p targets in DisGeNET and in Transcription Factor Targets. Online tool metascape https://metascape.org/ was used for enrichment analysis.
(TIF)

S3 Fig SKOV3 cells or 293FT cells were treated with KU60019 for 24 hours, the level of miR-1273g-3p in cells were determined using qPCR. (*p < 0.05; **p < 0.01).
(TIF)

S4 Fig KU60019-treated 293FT cells were treated with DGAT1 inhibitor or negative control for 24 hours, absorbance at 450 nm was recorded to determine cell viability. (TIF)

S5 Fig Correlation with the level of DGAT1 and tumor infiltrating lymphocyte in ovarian cancer tissues. (TIF)
