# Supplementary material for: KU60019 inhibits ovarian cancer progression by targeting DGAT1/has-miR-1273g-3p axis
Source: PLoS One. 2025 Jun 24;20(6):e0325213. doi: 10.1371/journal.pone.0325213 (PMC12186960; doi:10.1371/journal.pone.0325213)
Supplement: S1 Table — (DOCX) [file pone.0325213.s001.docx]

S1 Table KEGG analysis of DGAT1

| Term | Corrected *P*-Value | |
| --- | --- | --- |
| CYTOKINE_CYTOKINE_RECEPTOR_INTERACTION | | 0.00000514 |
| LEISHMANIA_INFECTION | | 0.00001181 |
| CHEMOKINE_SIGNALING_PATHWAY | | 0.00001181 |
| LYSOSOME | | 0.00023679 |
| NOD_LIKE_RECEPTOR_SIGNALING_PATHWAY | | 0.00074732 |
| FOCAL_ADHESION | | 0.00109749 |
| EPITHELIAL_CELL_SIGNALING_IN_HELICOBACTER_PYLORI_INFECTION | | 0.00165740 |
| APOPTOSIS | | 0.03831063 |
| ASCORBATE_AND_ALDARATE_METABOLISM | | 0.03831063 |
|  | |  |
