# Supplementary figures and images for: KU60019 inhibits ovarian cancer progression by targeting DGAT1/has-miR-1273g-3p axis
Source: PLoS One. 2025 Jun 24;20(6):e0325213. doi: 10.1371/journal.pone.0325213 (PMC12186960; doi:10.1371/journal.pone.0325213)

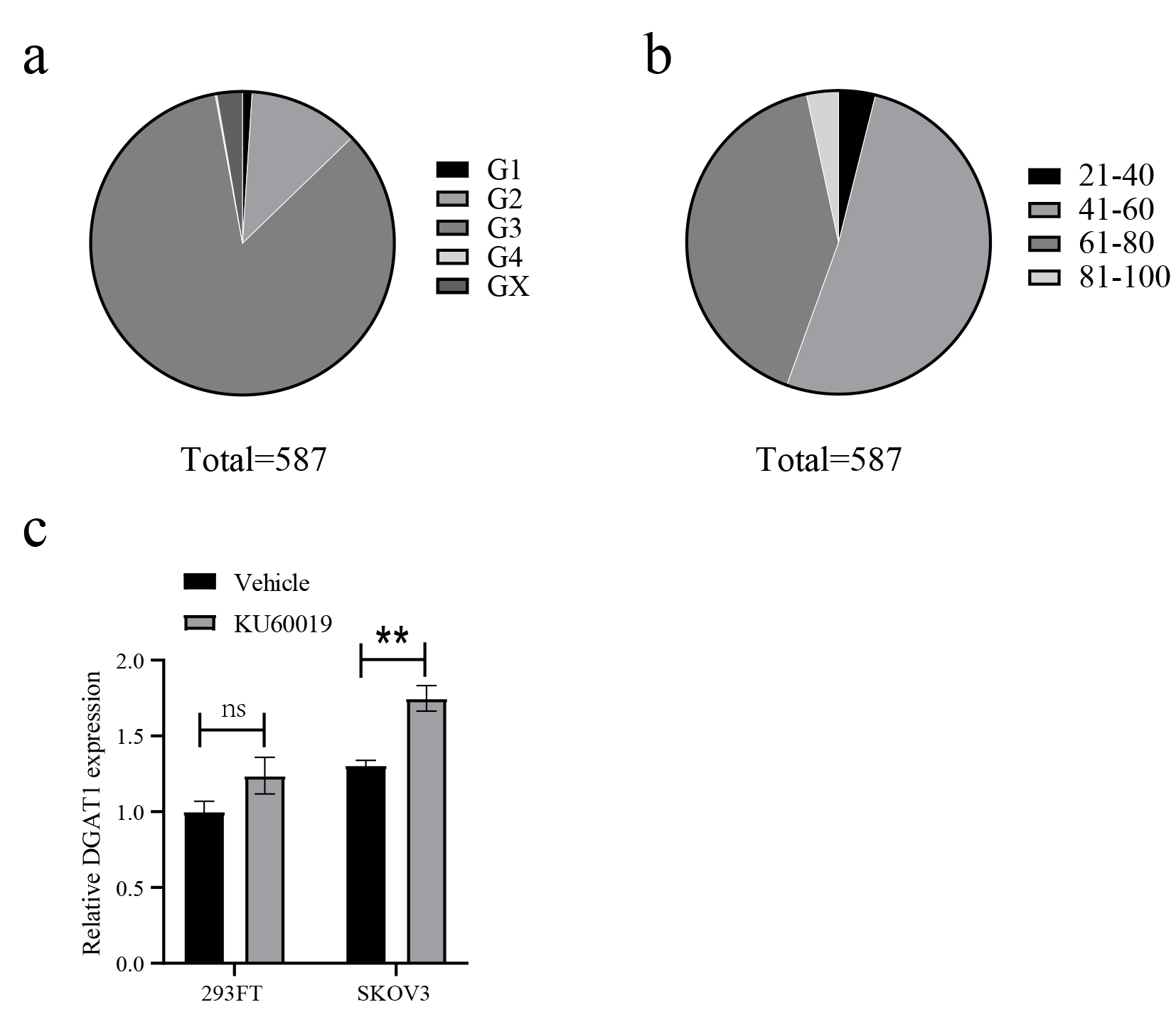

Supplement: S1 Fig — (a)The proportion of patients with different cancer stages. (G1, stage 1; G2, stage 2; G3, stage 3; G4, stage 4; GX, undetermined grade. n = 587). (b)The proportion of ovarian cancer patients in different age groups. Data was obtained from the TCGA. (n = 587). (c)SKOV3 cells or 293FT cells were treated with KU60019 for 24 hours, the level of DGAT1 in cells were determined using qPCR. (*p < 0.05; **p < 0.01). (TIF) [file pone.0325213.s002.tif]

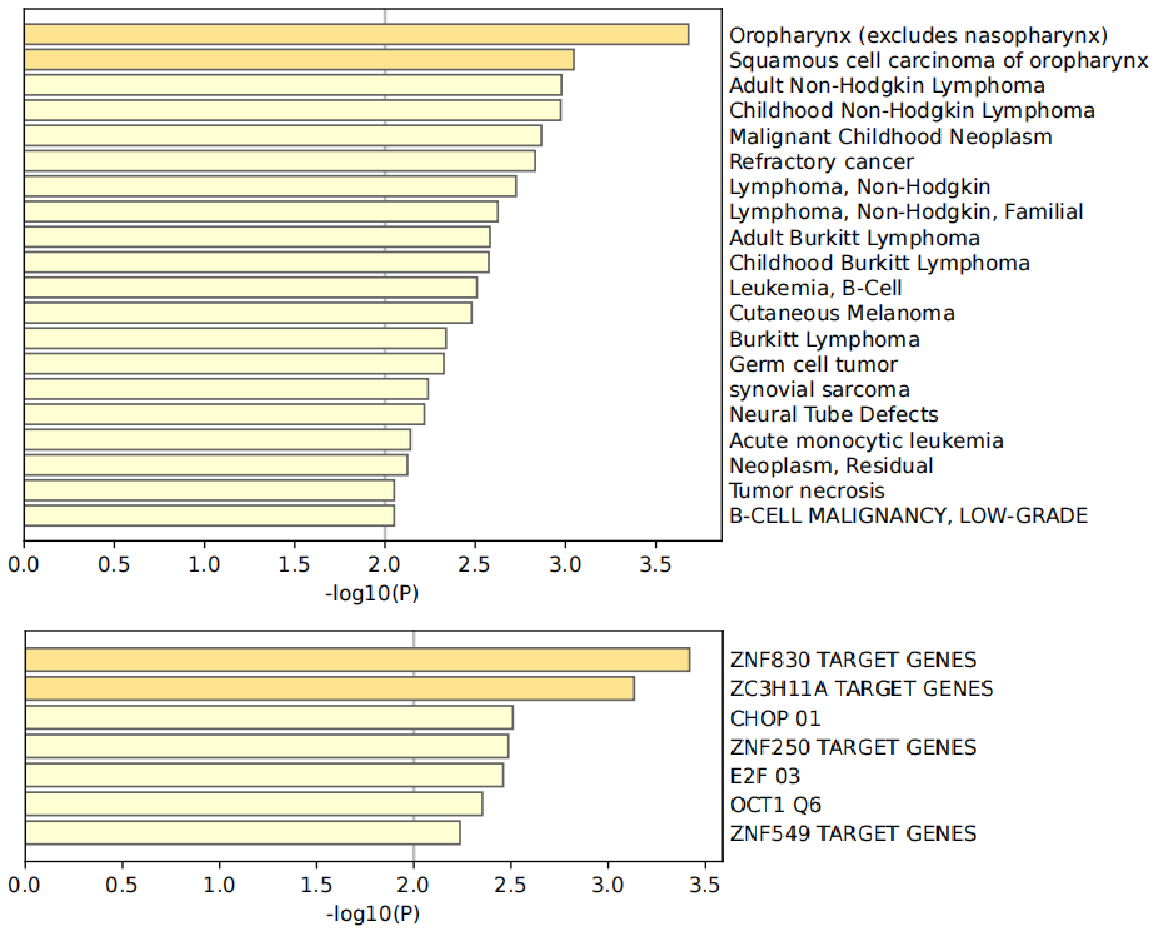

Supplement: S2 Fig — Online tool metascape https://metascape.org/ was used for enrichment analysis. (TIF) [file pone.0325213.s003.tif]

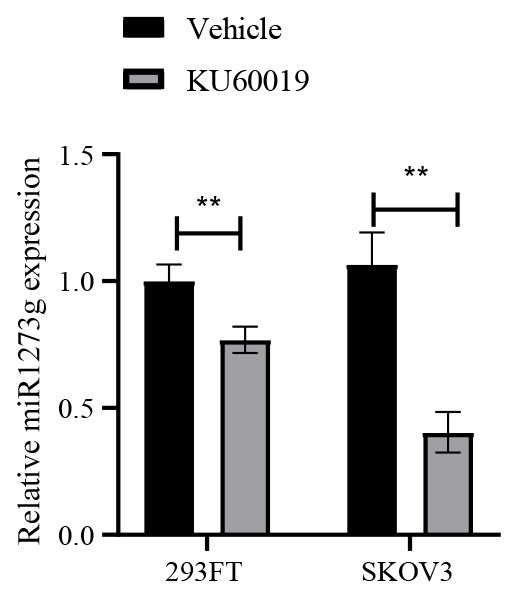

Supplement: S3 Fig — (*p < 0.05; **p < 0.01). (TIF) [file pone.0325213.s004.tif]

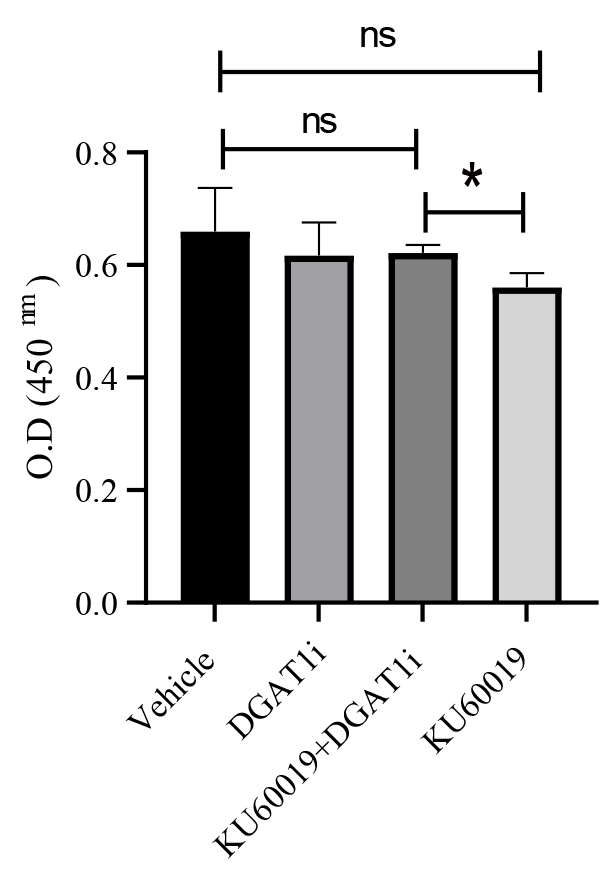

Supplement: S4 Fig — (TIF) [file pone.0325213.s005.tif]

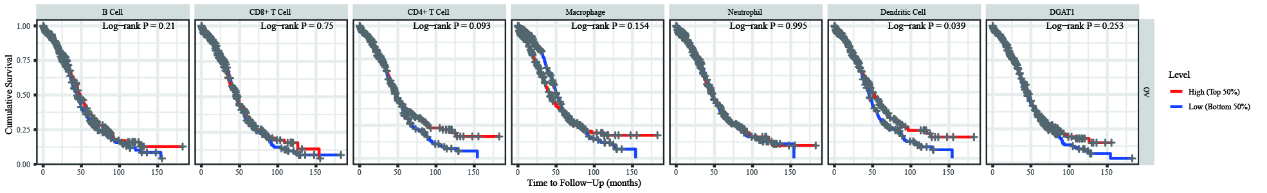

Supplement: S5 Fig — (TIF) [file pone.0325213.s006.tif]
